# 3D printing-assisted total hip arthroplasty and internal fixation for the treatment of fresh acetabular fracture and femoral head necrosis: A case report

**DOI:** 10.1097/MD.0000000000036832

**Published:** 2023-12-29

**Authors:** Yong Chen, Bin Zhang

**Affiliations:** a Department of Orthopedics Surgery, Central Hospital Affiliated to Shenyang Medical College, Shenyang, Liaoning, China.

**Keywords:** 3D printing technology, femoral head avascular necrosis, posterior wall acetabular fracture, total hip arthroplasty

## Abstract

**Rationale:**

Posterior wall comminuted fractures of the acetabulum are typically caused by high-energy trauma, and the complex anatomical structure of the acetabulum makes their treatment challenging. However, reports of the treatment of fresh acetabular fractures combined with femoral head necrosis are extremely rare.

**Patient concerns:**

A 57-year-old male, injured in a car accident, presented with right hip pain and limited mobility. At the age of 50, the patient was diagnosed with avascular necrosis of the right femoral head, experiencing right hip pain and a limp while walking, for which conservative treatment was initiated.

**Diagnosis:**

The patient was clinically diagnosed with fresh comminuted posterior wall acetabular fracture and late-stage femoral head necrosis.

**Interventions:**

We applied 3D printing technology and computer-assisted virtual surgical techniques for preoperative planning, simulated fracture reduction, and designed personalized bone plates and screws for fixation of the posterior wall of the acetabulum. A single-stage total hip arthroplasty was performed to treat femoral head necrosis.

**Outcomes:**

He began walking with the assistance of a walker 1 month after surgery, and at 6 months post-surgery, the acetabular posterior wall fracture had effectively healed, allowing the patient to return to work.

**Lessons:**

The application of 3D printing technology in acetabular internal fixation and total hip arthroplasty is helpful for fracture assessment, facilitates smooth surgery, promotes fracture reduction and healing, restores hip joint function, and ensures a high level of safety.

## 1. Introduction

Acetabular fractures are commonly caused by high-energy trauma and involve fractures within the joint. Due to the complex anatomical structure of the acetabulum, its deep location, and the proximity of blood vessels and nerves, treating such fractures presents significant challenges, making it a longstanding focus of clinical research. For displaced posterior wall acetabular fractures, our preferred approach is open reduction and internal fixation (ORIF).^[1]^ Total hip arthroplasty (THA) is an effective treatment method for advanced femoral head necrosis.^[2,3]^ However, there have been no reports on the treatment of patients simultaneously affected by fresh posterior wall acetabular fractures and femoral head necrosis. In this report, we describe a rare case of a patient with a fresh posterior wall acetabular fracture combined with advanced femoral head necrosis. We utilized 3D printing technology and computer-assisted virtual surgical techniques to conduct preoperative planning, simulate fracture reduction, and create 3D models for the patient.^[4]^ We designed a personalized bone plate for fixation of the posterior wall of the acetabulum, followed by a single-stage THA to treat femoral head necrosis, achieving favorable clinical outcomes.

## 2. Case report

**Figure 1.**
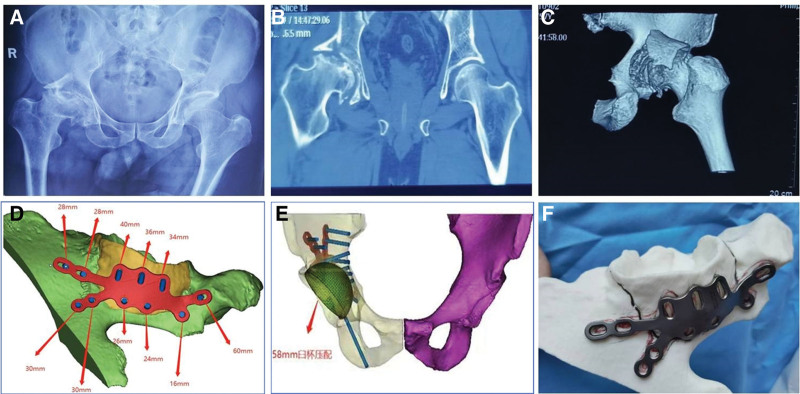
Preoperative X-ray, CT, and 3D printing design. (A–C) A fresh fracture of the posterior wall of the right acetabulum and femoral head necrosis. (D and E) Three-dimensional modeling in MIMICS software, designing personalized bone plate and evaluating the size of the acetabular cup. (F) 3D printing the acetabulum model and personalized bone plate.

The patient is a 57-year-old male who presented to our hospital after being struck by a vehicle, complaining of right hip pain and limited mobility. He arrived at our hospital approximately 9 hours after the incident. The patient had a previously good overall health status with no significant medical history, except for the discovery of avascular necrosis of the right femoral head at the age of 50, which caused right hip pain and a limp during walking. He had undergone conservative treatment for this condition. The right lower limb was approximately 1 centimeter shorter than the left lower limb. There was swelling in the right hip area, but the skin was intact. Local tenderness was observed in the right hip area, and percussion tenderness was positive along the longitudinal axis of the right lower limb. Due to severe pain upon passive movement, we were unable to measure the extent of right hip joint mobility. The right lower limb exhibited normal skin temperature and sensation, with no numbness. The right ankle joint had good mobility, and there was normal movement and sensation in all toes of the right foot. The dorsalis pedis artery in the right foot was palpable. Emergency X-rays revealed a discontinuity in the cortical bone of the right acetabulum with displaced bone fragments, flattening and localized collapse of the right femoral head, and a narrowing of the joint space (Fig. 1A). CT imaging showed a comminuted posterior wall fracture of the right acetabulum with displaced bone fragments, deformation of the right femoral head, and localized collapse (Fig. 1B and C). To better assess the patient acetabular condition preoperatively, we imported the patient preoperative CT data in DICOM format into MIMICS software for 3-dimensional modeling. We marked all bone fragments, simulated reduction of the fragments, designed a personalized bone plate, and pre-determined the bone plate information such as the placement position, screw entry point, screw lengths, and angles (Fig. 1D). Subsequently, we evaluated the depth of acetabular erosion and selected the size of the acetabular cup (Fig. 1E). Finally, we utilized 3D printing technology to create a 1:1 scale acetabular model and personalized bone plate (Fig. 1F).

**Figure 2.**
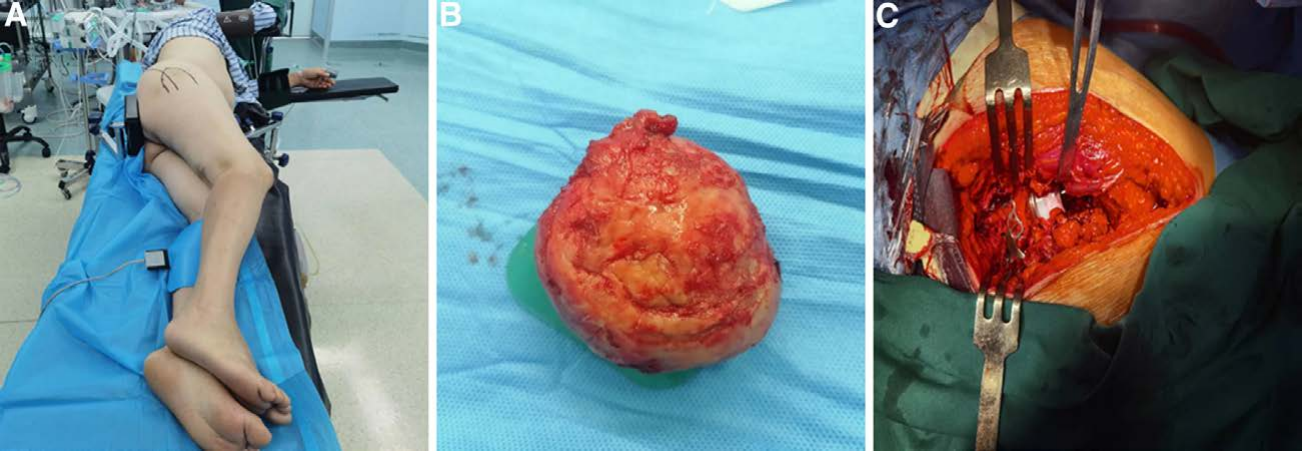
Patient position and intraoperative images. (A) A Kocher-Langenbeck (K-L) approach used as the surgical incision. (B) The femoral head is flattened and exhibits cartilage delamination. (C) Open reduction internal fixation and total hip arthroplasty.

After achieving satisfactory general anesthesia, the patient was placed in a left lateral decubitus position. A Kocher-Langenbeck approach was employed as the surgical incision (Fig. 2A). The procedure involved incising the skin, subcutaneous tissues, and fascial layers, followed by blunt dissection of the gluteus maximus muscle. At the point of the greater trochanter of the femur, the external rotator muscle group was transected. The surgery revealed a ruptured posterior joint capsule of the hip. Identification and protection of the sciatic nerve. A comminuted posterior wall acetabular fracture was identified, and the tissue at the fracture site was cleaned. The posterior wall of the acetabulum was reduced and temporarily fixed with Kirschner wires. A personalized bone plate was positioned on the posterior wall of the acetabulum and secured with screws. A compression screw was inserted vertically to the fracture line, ensuring secure fixation of the posterior wall of the acetabulum. Approximately 1.5 cm above the lesser trochanter, the femoral head was excised (Fig. 2B). Using an acetabular reamer, the acetabular cartilage was removed, with attention to achieving a 20-degree anteversion angle and a 45-degree abduction angle. A size 58 biological acetabular cup was implanted (Fig. 2C). Three cancellous bone screws were implanted in the outer superior quadrant. The femoral medullary canal was opened, a size 6 biological femoral stem was installed, and a standard ceramic head prosthesis was attached. After reduction, the hip joint exhibited good mobility without dislocation. Routine hemostasis procedures were performed, and the incision was closed layer by layer after irrigation.

**Figure 3.**
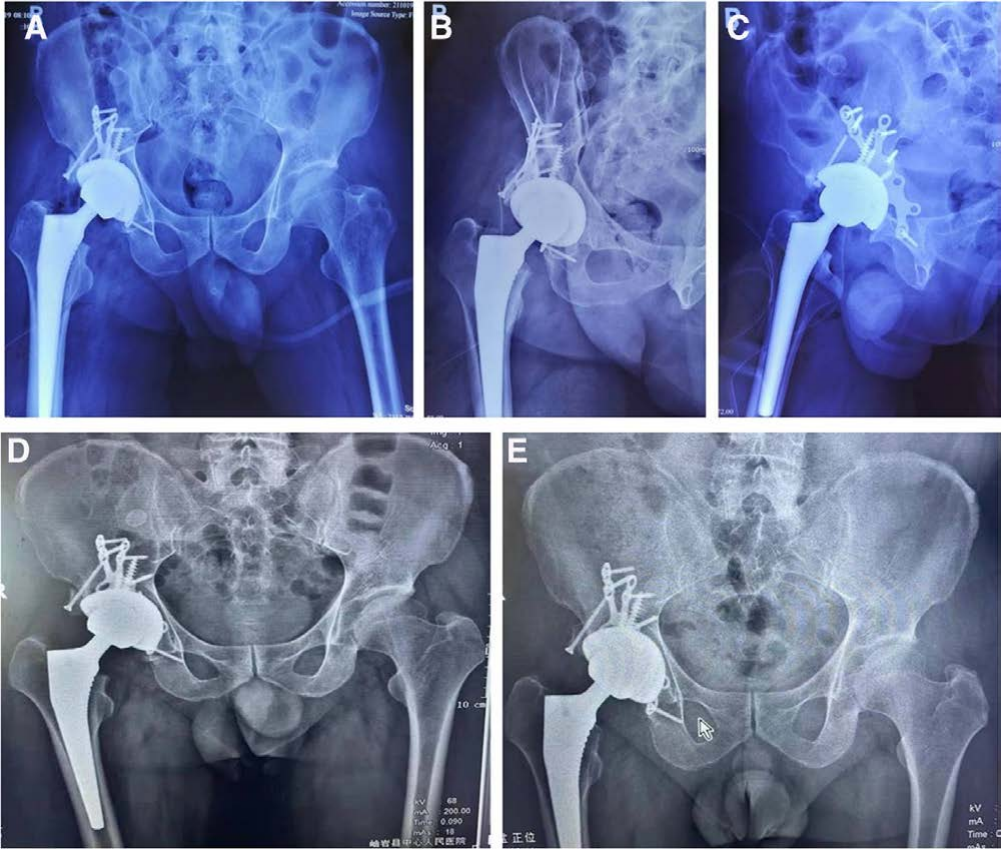
Postoperative X-ray images. (A–C) X-ray images of the patient right hip ORIF and THA 1 d postoperatively, including bilateral hip anteroposterior image, Obturator lateral image, and Iliac oblique image. (D) X-ray examination of the patient right ORIF and THA 1 mo postoperatively. (E) The X-ray shows that the posterior acetabular wall fracture has healed, and the prosthetic is in good position, 6 mo postoperatively. ORIF = open reduction and internal fixation, THA = total hip arthroplasty.

Following the surgery, the patient received treatment for pain management, facilitation of fracture healing, infection prevention, and prevention of deep vein thrombosis in the lower limbs. Postoperative X-rays demonstrated excellent reduction of the posterior wall acetabular fracture, proper positioning of the bone plate and screws, and favorable abduction and anteversion angles of the acetabular cup, with both femoral head rotation centers at the same level (Fig. 3A–C). On postoperative examination, both lower limbs were of equal length, and there was no numbness. The patient underwent functional exercises in bed postoperatively. At the 1-month follow-up, X-rays showed proper positioning of the implanted prosthesis (Fig. 3D), and the patient began walking with the assistance of a walker. By 6 months postoperatively, the patient could walk independently without the need for a cane. Follow-up X-rays confirmed the correct positioning of the prosthesis and complete healing of the posterior wall acetabular fracture (Fig. 3E). The patient reported minimal pain and was able to return to work.

## 3. Discussion

Posterior wall acetabular fractures are the most common type within the Judet-Letournel classification of acetabular fractures.^[5,6]^ Clinical cases of fresh acetabular fractures combined with femoral neck fractures or femoral head fractures have been observed,^[7,8]^ and in most clinical scenarios, internal fixation surgery is the preferred treatment option. There have also been reports of femoral head necrosis occurring as a complication following acetabular fracture internal fixation surgery,^[9]^ with the standard approach being salvage THA. However, there is a significant scarcity of reported cases involving fresh acetabular fractures combined with late-stage femoral head necrosis, and we lacked prior treatment experience for such patients.

There is currently significant controversy regarding the one-stage THA for fresh acetabular fractures.^[10]^ Multiple studies have shown that compared to isolated internal fixation, one-stage hip arthroplasty has a higher incidence of complications such as hip dislocation and infection.^[11,12]^ Because nonunion of the acetabular fracture can lead to loosening of the acetabular prosthesis, it is crucial to strictly adhere to surgical indications. For the case of the patient with a fresh posterior wall acetabular fracture in this report, our approach was ORIF. The standard Kocher-Langenbeck approach can successfully reduce the majority of posterior wall acetabular fractures, and fixation of the posterior wall fragment with tension screws and a bone plate is a standardized treatment approach for posterior wall acetabular fractures.^[13,14]^ However, in our case, the comminuted posterior wall fracture was accompanied by late-stage femoral head necrosis on the same side. ORIF alone could not alleviate the symptoms caused by femoral head necrosis. Therefore, the feasibility of performing THA on the basis of fixing the posterior wall of the acetabulum is worth exploring.

There are reports suggesting that in elderly patients with acetabular fractures accompanied by acetabular roof compression, hip dislocation, or femoral head injury, the outcomes of ORIF are often unsatisfactory, and one-stage THA may offer a better prognosis.^[15]^ Borg et al reported favorable outcomes of one-stage THA for the treatment of geriatric acetabular fractures.^[16]^ Kreder et al recommended that for patients older than 50 years with associated marginal compression and posterior wall comminution, consideration could be given to one-stage joint replacement.^[17]^

Although acetabular posterior wall fractures are considered simple fractures, the long-term outcomes are not optimistic, and post-traumatic arthritis and femoral head necrosis are common complications after acetabular fractures.^[18,19]^ These complications significantly impact the patient prognosis, leading to pain and functional impairment in the affected hip joint, ultimately necessitating THA. Therefore, for posterior wall fractures with multiple adverse prognostic factors, one-stage THA may be a prudent choice. While the patient in this case is relatively young at 57 years old, they presented with an acetabular posterior wall comminuted fracture and concomitant femoral head necrosis for 7 years, experiencing hip joint pain, limping, and limited mobility. X-rays and CT scans revealed femoral head necrosis with collapse. Thus, the patient met the criteria for THA.

We informed the patient and their family that there were 2 treatment options available. The first option was to undergo a one-stage procedure involving ORIF of the acetabular posterior wall fracture combined with THA. While this option could address both issues simultaneously, it would involve a longer surgical time and carry risks of infection and potential complications related to nonunion of the fracture. The second option involves performing an ORIF surgery for the acetabular posterior wall fracture first, and then, after the fracture has healed, proceeding with THA. While this approach is considered safer, it does require 2 separate surgeries. Additionally, there may be increased bleeding due to scar tissue formation around the incision sites, and it may result in higher overall costs. After careful consideration, the patient and their family believe that the first option is more favorable. They are willing to accept the surgical risks associated with one-stage surgery to reduce the trauma, stress, and economic burden of undergoing 2 separate surgeries.

We respect the patient and their family preferences and adhere to the principles of Enhanced Recovery After Surgery to shorten the surgical duration, reduce blood loss, and minimize infection risk. We utilized 3D printing technology and computer-assisted virtual surgical techniques for preoperative planning in the case of the posterior wall fracture. Given the irregular bone surface of the acetabular posterior wall and the presence of surrounding blood vessels and nerves, we designed a more customized bone plate for optimal fit to the posterior wall. This design also included planning the locations for screw holes and measuring the angles and lengths of the implanted screws. We assessed the depth of acetabular wear and determined the appropriate size for the acetabular cup. We print a 1:1 model of the acetabulum and create a personalized bone plate. These steps facilitated communication with the patient and their family, involving them in the surgical discussion, which helped reduce patient anxiety and stress. Additionally, it enabled the surgical team to better understand the specifics of the acetabular fracture, select suitable prosthetics, confirm the implantation sites, prevent screws from entering the joint cavity, and avoid damaging surrounding blood vessels and nerves. It also helped determine the appropriate angles for the acetabular cup to avoid contact with the screws. Preoperative simulated surgery enhanced the chances of a successful operation and reduced the risk of surgical complications.

However, 3D printing technology also has certain limitations. The accuracy of bone signal image segmentation by software engineers and the proficiency of radiology technicians in collecting CT image data determine the extent of the differences between 3D models and real bone structures. Additionally, 3D printing cannot achieve complete differentiation between soft tissues such as muscles, ligaments, and bones. This limitation results in the designed bone plate not being able to perfectly conform to the irregular bone surface.

Pure internal fixation for the treatment of acetabular fractures alone cannot alleviate the symptoms caused by femoral head necrosis. THA is a better choice for this patient, and the patient did not experience any significant intraoperative or postoperative complications. After the surgery, his pain has reduced, hip joint function has recovered well, and he has reintegrated into society. The patient is satisfied with the treatment, and now that the fracture has healed, he continues to undergo follow-up. In summary, the application of 3D printing technology in THA and acetabular internal fixation is helpful for fracture assessment, facilitates smooth surgery, promotes fracture reduction and healing, restores hip joint function, and ensures a high level of safety. This case report aims to provide orthopedic surgeons with an option for treating fresh acetabular fractures complicated by femoral head necrosis.

## Author contributions

**Conceptualization:** Bin Zhang.

**Data curation:** Bin Zhang.

**Methodology:** Bin Zhang.

**Supervision:** Bin Zhang.

**Validation:** Yong Chen.

**Visualization:** Yong Chen.

**Writing – original draft:** Yong Chen.

**Writing – review & editing:** Yong Chen.
